# How Bodies and Voices Interact in Early Emotion Perception

**DOI:** 10.1371/journal.pone.0036070

**Published:** 2012-04-30

**Authors:** Sarah Jessen, Jonas Obleser, Sonja A. Kotz

**Affiliations:** 1 Minerva Research Group “Neurocognition of Rhythm in Communication”, Max Planck Institute for Human Cognitive and Brain Sciences, Leipzig, Germany; 2 Cluster of Excellence “Languages of Emotion”, Free University of Berlin, Berlin, Germany; 3 Max Planck Research Group “Auditory Cognition”, Max Planck Institute for Human Cognitive and Brain Sciences, Leipzig, Germany; University of California San Diego, United States of America

## Abstract

Successful social communication draws strongly on the correct interpretation of others' body and vocal expressions. Both can provide emotional information and often occur simultaneously. Yet their interplay has hardly been studied. Using electroencephalography, we investigated the temporal development underlying their neural interaction in auditory and visual perception. In particular, we tested whether this interaction qualifies as true integration following multisensory integration principles such as inverse effectiveness. Emotional vocalizations were embedded in either low or high levels of noise and presented with or without video clips of matching emotional body expressions. In both, high and low noise conditions, a reduction in auditory N100 amplitude was observed for audiovisual stimuli. However, only under high noise, the N100 peaked earlier in the audiovisual than the auditory condition, suggesting facilitatory effects as predicted by the inverse effectiveness principle. Similarly, we observed earlier N100 peaks in response to emotional compared to neutral audiovisual stimuli. This was not the case in the unimodal auditory condition. Furthermore, suppression of beta–band oscillations (15–25 Hz) primarily reflecting biological motion perception was modulated 200–400 ms after the vocalization. While larger differences in suppression between audiovisual and audio stimuli in high compared to low noise levels were found for emotional stimuli, no such difference was observed for neutral stimuli. This observation is in accordance with the inverse effectiveness principle and suggests a modulation of integration by emotional content. Overall, results show that ecologically valid, complex stimuli such as joined body and vocal expressions are effectively integrated very early in processing.

## Introduction

Body expressions and vocalizations play an important role in communication. We can readily determine from either modality someone's gender [1], emotion [2], [3], or how familiar a person is [4], [5]. Crucially, these sources of information are not independent: both are rooted in biological motion. While body expressions per definition are biological motion, vocalizations are generated by our vocal tract and strongly influenced by body posture, making them a product of biological motion. Furthermore, both provide closely time–locked and congruent information.

Not surprisingly, early interactions of body and vocal expressions are reliably observed [6], [7], and an interaction has also been reported between body motion and music produced by the very body motion [8], [9]. However, do these interactions truly reflect integration [10]? Various approaches have been suggested to address this question. One popular method, especially in the investigation of audiovisual emotion perception, is the use of mismatch paradigms, in which violation responses can be observed when the two modalities provide conflicting information [11], [12]. While these studies suggest an integration of facial and vocal information, they can only indirectly infer integration from incongruent responses. Additionally, in an ecologically valid context, congruency between modalities is far more common than incongruency.

A second approach to quantify multisensory integration is the comparison of an audiovisual condition to the sum of the two unimodal conditions, potentially resulting in either sub- or superadditivity [13], [14]. However, the reliability of this criterion has recently been criticized [15], [16]. An alternative way to test multisensory integration is the inverse effectiveness (IE) paradigm, which states that interaction effects should be largest if a stimulus is unimodally least effective [10]. Originally developed in neurophysiological recording [10], [17], [18], this paradigm has been extended to functional magnetic resonance imaging (fMRI) studies [19], [20]. Recently, Senkowski and colleagues [21] were able to demonstrate inverse effectiveness also in event-related potentials (ERPs). Using very simple visual and auditory stimuli, they report enhanced multisensory interactions for low intensity stimuli within 60 ms after stimulus onset. Furthermore, behavioral studies show that IE can be observed in the integration of dynamic emotional stimuli [22]. In the present study, we seek to extend these previous findings by investigating the neural underpinnings of multisensory perception in an ecologically valid communicative context.

Such a communicative context comprises facial, bodily, and vocal expressions, however, most previous studies investigating multisensory communication have focused on the interplay between facial and vocal [11], [23] or facial and bodily expressions [24]. While we were able to demonstrate an early interaction between facial, body, and vocal expressions in a previous study [7], it cannot be ruled out that the reported results were mainly driven by the processing of facial expressions. To focus specifically on the body–voice interaction, in the current study we blurred facial expressions contained in the stimulus material to avoid any influence of facial information. In contrast to previous studies using incongruent body and vocal expressions [6], [25], we further investigate a possible integration in a congruent setting.

Hence, we addressed two main questions. (i) Can an early interaction between voice and body be observed in ecologically valid stimulus set-ups? If so, does this interaction result in facilitated processing, as has been previously described for audiovisual interactions [26], [27]? (ii) Does such an early interaction follow the inverse effectiveness principle, suggesting multisensory integration [20]?

While this interaction is important for many communicative settings, an especially striking one is emotional communication. Correctly identifying others' emotions is of high relevance, and perceiving emotions via multiple modalities provides a strong processing benefit [28], [29]. Emotion perception therefore offers an ideal example to examine the interaction between body expressions and vocalizations.

We recorded the electroencephalogram (EEG) to investigate early emotional and sensory processes. To assess the impact of visual on auditory processing, we focused on the auditory N100, an ERP component robustly reflecting this impact [30] and indicating facilitated processing in shorter peak latencies [31] and reduced peak amplitudes [30], [31]. As the sound onset in the unmanipulated, natural videos does not coincide with the video onset, computing ERPs in relation to the video onset was not of interest to the present study. In addition, the information contained in the video material is unfolding over the course of several seconds with no fixed event to allow for the computation of ERPs measuring visual processing. To circumvent these problems and assess non-phase-locked interactions between visual and auditory processing, we analyzed oscillatory changes in EEG power. In particular, a suppression of the beta–band (15–25 Hz) [32], [33] is thought to reflect the processing of biological motion such as body movements. We therefore flanked the N100 ERP-analysis with a time–frequency-analysis. To further support the assumption that the observed beta–band effects indeed reflect the processing of biological motion, we conducted a source localization of beta–band changes based on the results of the time–frequency-analysis.

## Materials and Methods

### Participants

Twenty-four native German speakers (12 female), who had not taken part in the study reported in Jessen and Kotz (2011), participated in the current study (mean age 24.7 years, SD (standard deviation) = 2.9 years). All were right-handed, had normal or corrected-to-normal vision, and did not report any hearing impairments. They were compensated financially for their participation, and gave written informed consent prior to the experiment. The study was approved by the ethics committee at the University of Leipzig.

### Stimuli and Design

**Figure 1:**
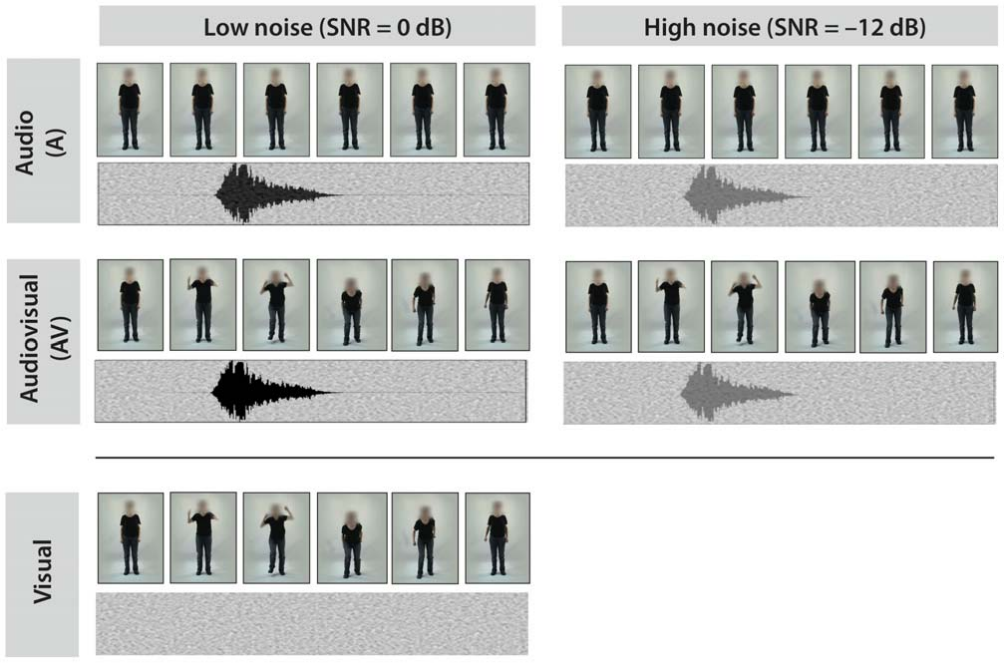
Example of the stimulus material. Six frames of a video clip of a female actress depicting “anger” are shown in the five conditions used in the experiment. To test for inverse effectiveness, we compared the difference between audiovisual and auditory stimuli under high and low noise conditions ((AV-A)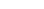 vs. (AV-A)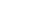). Additonally, we recorded responses in a purely visual condition (bottom row).

We used video clips containing emotional body expressions as well as recordings of emotional interjections (“ah”, “oh”, and “mh”) [34] in three different affective states: “anger”, “fear”, and “neutral” (see Figure 1 for an example). All three states were video recorded from four different semi-professional actors; 2 women (24 and 41 years old) and 2 men (30 and 48 years old) and each emotion was expressed vocally by the interjections. We recorded several takes of each emotion. Based on a prior rating study (see below) we selected 10 items for each condition, amounting to 360 stimuli in total. In addition to the video clips, for each actor we extracted one still image from a recording prior to the movement onset. This still image, in which the actor was standing in a neutral position, was shown on the screen during all auditory stimuli produced by the respective actor. Further details about the recording of the stimulus material can be found elsewhere [7]. In order to assess possible differences in the motion content of the video clips, we calculated the amount of motion contained in each video by computing the average luminance change [7], [35]. Neutral and fearful videos did not differ regarding the amount of motion, while anger videos showed a slightly lower motion content. Therefore, neutral videos did not differ systematically from emotional ones in the amount of motion contained.

In order to investigate the perception of body expressions while controlling for facial expressions, the faces of the actors were blurred using the software Motion 3.0.2 (Apple Inc.), rendering the facial expressions unrecognizable. In a pilot study 16 participants were asked to categorize the emotion expressed by the actors in these videos; the intended emotion was recognized with a mean accuracy of 96.2% (SE (standard error) = 0.8). All auditory stimuli were embedded in white noise using Matlab 7.7.0 (The MathWorksInc, Natick, MA, USA). In a second pilot study, the auditory stimuli were presented to 15 participants at different signal-to-noise ratios (SNRs): 0 dB, −6 dB, −12 dB, −18 dB, and −24 dB. The participants were asked to judge the expressed emotion. The SNR of −12 dB resulted in a decrease in performance but emotions were still reliably recognized above chance (average recognition accuracy of 81.1%, SE = 2.2). This ratio was chosen as the high-noise condition in the EEG experiment. As the low-noise condition, a signal-to-noise ratio of 0 dB was used (average recognition accuracy of 88.2%, SD = 1.6). We decided to use a low-noise rather than a no-noise condition in order to minimize the physical difference between the two noise levels.

Stimuli were presented in five conditions (Figure 1). In two auditory conditions, interjections were presented at an SNR of 0 dB (audio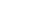) or −12 dB (audio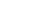) while a static picture of the respective actor was shown on the screen, and thus no visual dynamic emotional information was conveyed. In the two audiovisual conditions, interjections were presented at the same SNRs, but this time accompanied by the matching video (audiovisual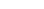 and audiovisual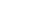, respectively). Finally, in the visual condition, videos were presented accompanied by a sound file containing white noise but no interjection. In each of these conditions, 24 angry, 24 fearful, and 24 neutral stimuli were presented, amounting to a total of 360 stimuli. The distribution of actors and interjections was counterbalanced across all conditions.

### Data Acquisition

Participants sat comfortably in a dimly lit sound-shielded chamber at a distance of approximately 120 cm from the monitor. Videos were presented at a visual angle of 8.86. Auditory stimuli were presented via headphones (SONY Stereo Headphones, MDR-XD100) at the same level of loudness for all participants. Before the experiment, the participants completed a short training that consisted of 10 trials and contained items not used in the actual experiment. A trial started with the presentation of a fixation cross (1 second), followed by the presentation of the stimulus which lasted on average 4.37 seconds (SD = 1.03). After each stimulus, the participants judged the previously seen emotion in a forced-choice task with the options “anger”, “fear”, and “neutral” using the three left-most keys of a 4-button response box. Half of the participants responded with the index-, middle-, and ring finger of the right hand, while the other half used their left hand. Furthermore, the response assignment was also counter-balanced across all participants. After 3 seconds, or as soon as the participant responded, an inter-trial-interval of 2 seconds started, during which the participants saw a gray screen. The participants were instructed to avoid eye blinks during the trial and use the inter-trial-interval to blink. After 20 trials participants took a short, self-paced break, leading to a total of 18 blocks. The experiment was programmed using the Presentation software package (Neurobehavioral Systems, Inc.).

We recorded from 64 Ag-AgCl electrodes according to the modified 10–20 system [36]. The sternum served as ground, the left mastoid as reference. Electrodes were mounted in an elastic cap (Electro-Cap International, Eaton, OH, USA), impedances were kept at less than 5 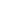 and the signal was bandpass-filtered only between DC and 140 Hz and recorded using the BrainVision Recorder software (Brain Products GmbH, Munich, Germany).

### Data Analysis

As the task was mainly designed to direct the participants' attention to the stimulus material, we do not focus on the behavioral results. Nevertheless, we computed *d′* as the discrimination ability between each pair of emotions (anger vs. fear, anger vs. neutral, fear vs. neutral), separately for the five modality conditions [37]. On the resulting values, a repeated–measures ANOVA with the factors emotion–contrast (anger vs. fear, anger vs. neutral, fear vs. neutral) and modality (audio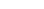, audio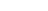, audiovisual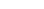, audiovisual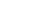, visual) was computed.

For computing ERPs, the data was re-referenced offline to linked mastoids, and filtered with a 1–30 Hz bandpass filter. Trials containing EOG artifacts larger than 30 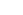 were rejected automatically, and the resulting data were inspected visually to remove additional artifacts. Two participants had to be excluded from further analysis due to excessive artifacts. We excluded trials in which the emotional content was not correctly identified, and averaged the remaining trials over a length of −200 to 2000 ms in relation to the sound onset. As accuracy rates varied between the different conditions, the number of trials averaged differed. In order to ensure the results were not confounded by a varying signal-to-noise ratio resulting from the varying number of trials (due to artifact rejection and accuracy differences), we computed the same ERP analyses described below with only 60% of the trials per condition, and thus with the same number of trials. We obtained the same results as with the full number of trials, and hence in the following report results with all correct trials per condition.

We conducted two separate analyses of the ERPs, one considering the peak latency in a time-window of 100–230 ms after sound onset, and one considering the amplitude at the peak of the component. Based on the visual inspection of the topographic distribution of the ERP-responses we defined a fronto-central region of interest (FC3, FCz, FC4, C3, C1, Cz, C2, C4). For both, amplitude and peak latency, we computed a repeated–measures ANOVA with the factors emotion (anger, fear, neutral), modality (audiovisual, audio), and noise-level (high, low), and Student's t-tests for a step-down analysis of interactions. The visual condition was not included in this specific analysis of the auditory evoked response, as no visual ERP was expected at this time point. A Greenhouse-Geisser correction [38] was applied to contrasts including more than two conditions. Furthermore, the results of the Student's t-tests were corrected for multiple comparisons using the Bonferroni-Holm-method [39], resulting in corrected alpha-thresholds of 0.05, 0.025, and 0.017 for an initial p-value of 0.05. Effect sizes for ANOVAs are given in 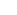, while effect sizes for t-tests are given in 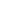 [40].

To analyze the data in the frequency domain, we re-referenced the data to the average of all electrodes and again excluded trials containing EOG artifacts larger than 30 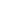. Furthermore, a bandpass filter was applied, ranging from 0.1–100 Hz. For each trial, we computed a time–frequency representation in a time-window of −1000 to 1000 ms relative to the sound onset using the Matlab toolbox FieldTrip [41]. We used Morlet's wavelets [42] with a time–frequency relation of m = 7, and calculated condition and subject-specific average time–frequency representations. Changes in spectral power were computed relative to a baseline of −500 to 0 ms before the video onset. Based on a previous study by Perry et al. [33] as well as our own previous work using comparable multimodal stimulation [7], we focused on the low beta range (15–25 Hz). Based on the visual inspection of the data, we chose a time-window of 200–400 ms for further analysis. In this time-window, the strongest difference in beta-suppression was observed in the emotion conditions. In addition, we computed a running “effects of interest” F-test checking for any condition effect at each time-bin between 0 and 1000 ms in relation to sound-onset. Only in a time-window of 200–400 ms, robust effects were observed. We extracted the average power change across the respective time-window for two groups of electrodes, a central group (C3, C1, Cz, C2, C4) and an occipital group (O1, Oz, O4). While central electrodes have been commonly used to measure changes in mu and beta rhythm, occipital electrodes have been used to measure sensory processing [33]. On these average power changes we performed the same statistical comparisons as described in the ERP section, that is, a repeated–measures ANOVA with the factors emotion (anger, fear, and neutral), modality (audio, audiovisual), and noise-level (high, low). In order to contrast the visual and the audiovisual conditions, we computed an additional repeated–measures ANOVA with the factors emotion (anger, fear, and neutral) and modality (visual, audiovisual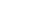 and audiovisual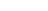). Again, we used Greenhouse-Geisser correction [38] when appropriate, the Bonferroni-Holm-method to correct for multiple comparisons [39], and report effect sizes for ANOVAs in 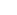 and for t-tests in 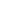 [40]. To ensure the observed power-suppression is indeed induced, we furthermore computed the inter-trial coherence [43], irrespective of condition.

In order to localize the source of the observed changes in beta-band power, we used dynamic imaging of coherent sources (DICS) [44], a localization algorithm particularly suited for localizing sources of oscillatory activity. We followed a protocol suggested in various previous studies [45], [46]: We repeated the time–frequency-analysis using a Hanning multi-taper approach, this time focusing on 20 Hz with a 5 Hz smoothing in a time-range of 200–700 ms after sound onset, thereby selecting the time-window chosen above for the statistical analysis but extending it to have a sufficient length to obtain a reliable estimation and using a multitaper frequency transformation. The source localization was conducted using electrode locations for the modified 10–20-system and a standard MRI template. In a first analysis step, we contrasted the overall beta activity (across all conditions) to the beta activity in the baseline using a one-sampled t-test in order to identify brain structures most likely generating the overall beta power. In a second analysis, we specifically aimed at localizing the condition differences in beta-suppression, and thus to compare the emotion conditions (anger and fear) to the neutral condition, collapsed across both audiovisual conditions irrespective of noise level, using a one-way within-subject ANOVA. While the source localization was computed using FieldTrip [41], the statistical comparisons as well as the visualizations were calculated in SPM8 (Wellcome Department of Imaging Neuroscience, London, UK).

## Results

### Behavioral Results

**Figure 2:**
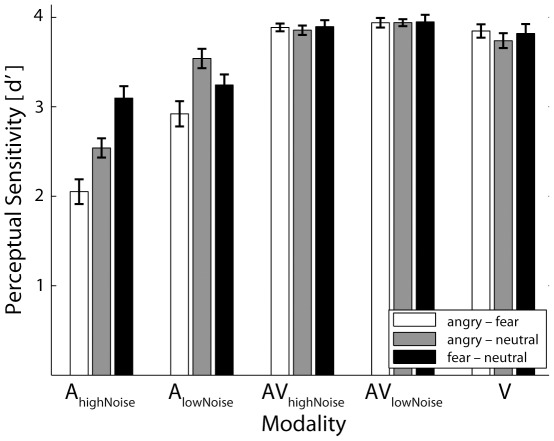
d′ of behavioral responses. Perceptual sensitivity for distinguishing anger from fear (anger–fear), anger from neutral (anger–neutral), and fear from neutral (fear–neutral) is depicted separately for each modality condition. Both auditory conditions show a significantly lower sensitivity than either audiovisual or the visual condition. Furthermore, lower d′ values are observed for A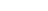 compared to A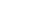.

Emotions were recognized above chance in all conditions (accuracy for the different modality conditions: audio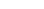: 92.42% (SD = 5.53); audio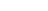: 82.77% (SD = 8.28); audiovisual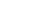: 98.48% (SD = 2.99); audiovisual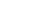: 98.11% (SD = 2.59); visual: 94.19% (SD = 9.77)). Auditory stimuli, irrespective of noise level, were differentiated worse than either audiovisual or visual stimuli (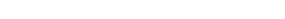) (see Figure 2). Furthermore, participants showed lower perceptual sensitivity for auditory stimuli at high noise levels compared to low noise levels (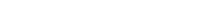). An interaction with the factor emotion (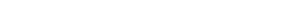) shows that for high a well as low noise levels in the auditory condition, participants performed worst at distinguishing anger from fear (high noise: 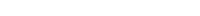, low noise: 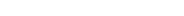, both compared to the next-worst distinction). While in high noise levels, the discrimination is worse between anger and neutral compared to fear and neutral (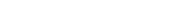), this was reversed in low noise levels (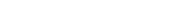).

### ERP Latency

**Figure 3:**
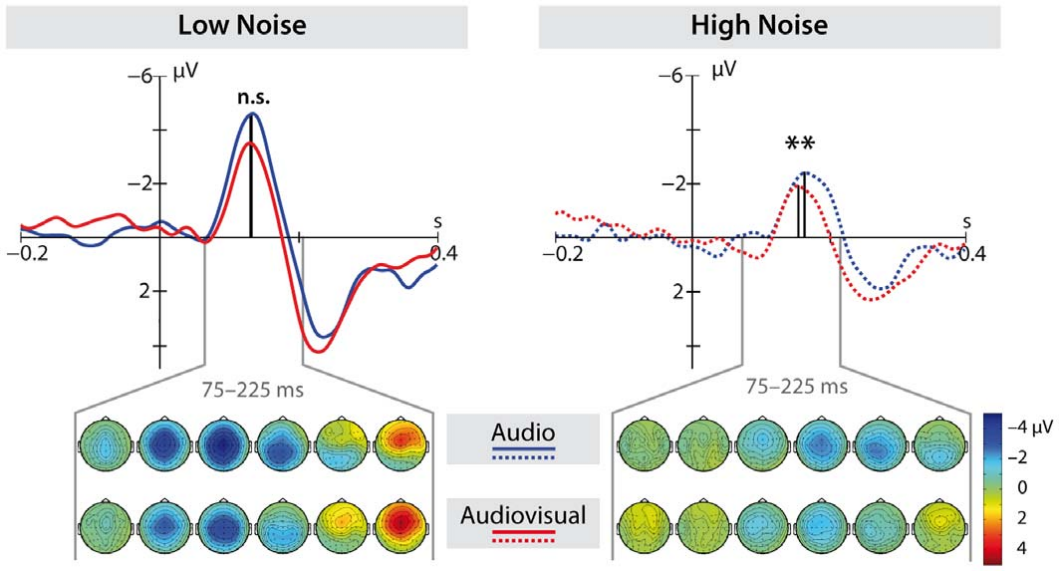
Multisensory interaction in high and low noise. Event-related potentials (averaged over FC3, FCz, FC4, C3, C1, Cz, C2, C4) in response to the auditory onset are depicted in the low-noise condition (left panel, bold) and in the high-noise condition (right panel, dotted). In red, the response in audiovisual conditions can be seen, while the response to auditory conditions is shown in blue. Earlier N100 peak-latencies can be observed in the high noise condition but not in the low noise condition (n.s. = not significant, 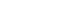). In the lower part, topographical maps of the auditory condition (top row) and the audiovisual condition (bottom row) are depicted in steps of 25 ms, starting at 0.075 ms after auditory onset.

**Figure 4:**
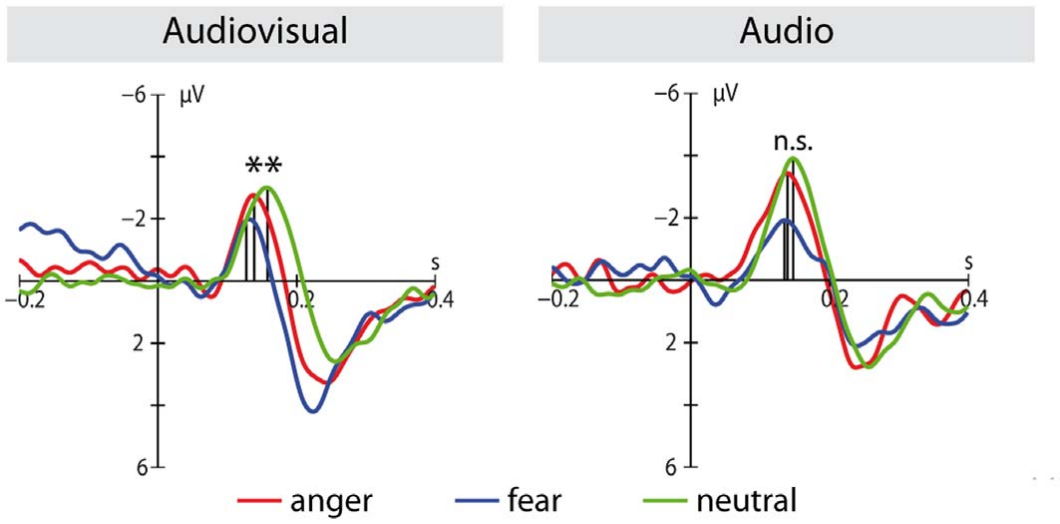
Emotion processing in the N100. Event-related potentials (averaged over FC3, FCz, FC4, C3, C1, Cz, C2, C4) in response to the auditory onset in the audiovisual condition (left panel) and the auditory condition (right panel) are shown for the different emotions (red = anger, blue = fear, green = neutral). Only in the audiovisual condition, a reduction in N100 peak-latency is observed (n.s. = not significant, 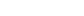).

We observed shorter N100 latencies for audiovisual compared to auditory stimuli (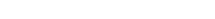), as well as for low noise compared to high noise (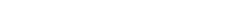). As can be seen in Figure 3, an interaction between these two factors (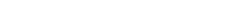) shows that these effects are mainly driven by a speed–up of the audiovisual compared to the audio condition at high noise levels (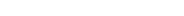), while there was no difference at low noise levels. Irrespective of the noise level, we found a latency reduction for emotional compared to neutral stimuli (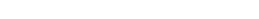) (see Figure 4). As revealed by an interaction between modality and emotion (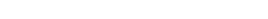), this reduction only occurred in the audiovisual condition (anger vs. neutral: 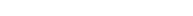, fear vs. neutral: 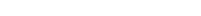).

In order to test the robustness of the obtained results, we furthermore conducted a jackknife analysis [47]. In brief, the peak latency was tested not on *n* single-subject averages, but on *n* grand averages of *n*–1 subjects. When the resulting t- or F-test statistics were adjusted by formulas provided in [47], all significant effects described above were also observed in the jackknife analysis, in particular the interaction between modality and noise (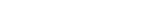) and the interaction between emotion and modality (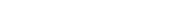).

### ERP Amplitude

Smaller N100 amplitudes were observed for audiovisual compared to auditory stimuli (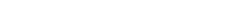) as well as for high noise compared to low noise stimuli (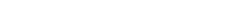). Furthermore, we found a smaller N100 amplitude for fearful stimuli compared to both, angry (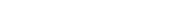) and neutral stimuli (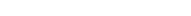).

### Time–Frequency Results

**Figure 5:**
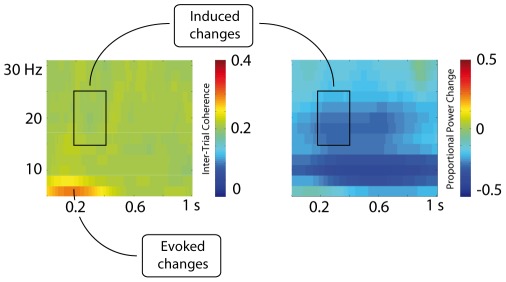
Intertrial coherence and power changes across all conditions. In the left panel, the intertrial coherence irrespective of condition is depicted. While a strong coherence can be seen shortly after sound onset in the range below 10 Hz, this is not the case for the time-window used to investigate the beta-suppression, indicated by the black frame. In the right panel, power changes are shown, again irrespective of condition.

**Figure 6:**
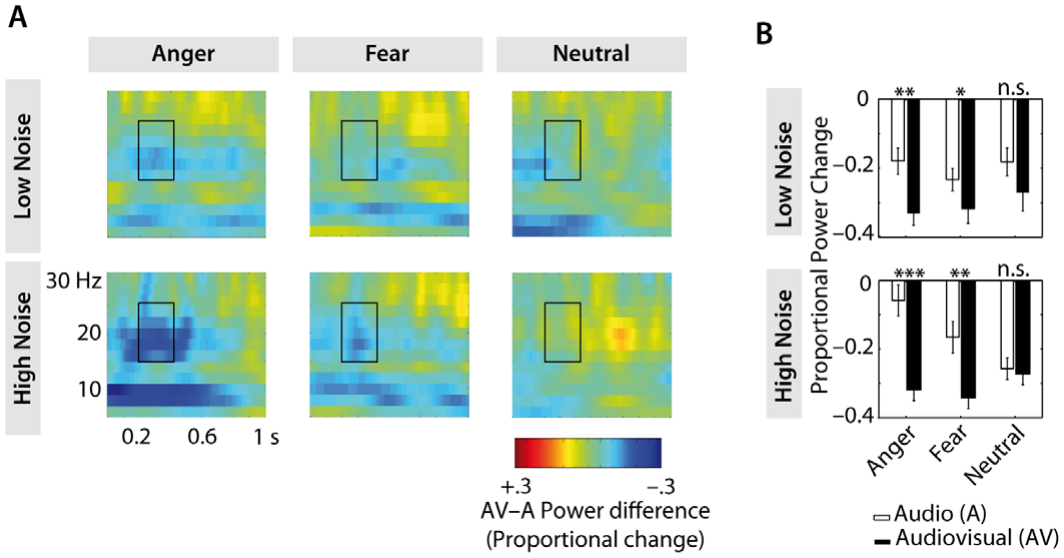
Beta-suppression in different conditions. Power changes relative to baseline are shown for one second after auditory onset and in a frequency range of 5–31 Hz (6A). Each plot depicts the difference in spectrum between the audiovisual and the auditory condition (AV-A). In the top row, differences in the low noise condition are shown, while in the bottom row, differences in the high noise condition are depicted (in both cases from left to right: anger, fear, neutral). In 6B, mean values for the auditory as well as the audiovisual condition are depicted for the range marked by the black frame (200–400 ms, 15–25 Hz). While stronger suppressions for audiovisual compared to auditory conditions can be seen in both emotion conditions, these differences are larger in the high-noise condition than in the low-noise condition (n.s. = not significant, 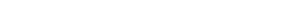).

As can be seen in Figure 5, the analyzed time-window shows a comparably low inter-trial coherence (around 0.2; phase-locking ranging from 0 to 1) in the beta-band, demonstrating that power changes are indeed induced rather than phase-locked [48]. At all frequency ranges and in all electrode groups we observed a stronger suppression for audiovisual compared to auditory stimuli (central: 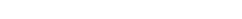; occipital: 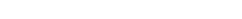) (Figure 6). Emotional processing did affect the suppression of oscillatory activity differentially dependent on the electrode location. Only at occipital electrodes (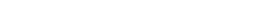) a stronger suppression was observed for fearful compared to neutral video clips (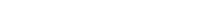), as well as for angry compared to neutral video clips (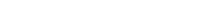). At both, central and occipital electrodes, we observed an interaction between the factors emotion and modality (central: 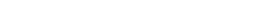; occipital: 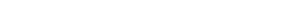), showing a stronger suppression for audiovisual compared to auditory stimuli only for angry (central: 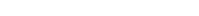; occipital: 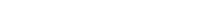) and fearful stimuli (central: 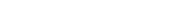; occipital: 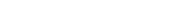). Finally, we observed a three-fold interaction between emotion, noise, and modality at central electrodes (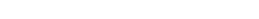), as depicted in Figure 6. Only at high levels of noise (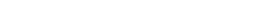), we observed a larger suppression for audiovisual compared to auditory stimuli for angry (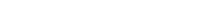) and fearful (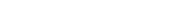) stimuli.

In the second model we included the visual and both audiovisual conditions and found no modality differences. As in the previous comparison, emotional stimuli resulted in a larger suppression at occipital electrodes (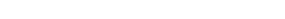; anger vs. neutral: 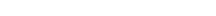, fearful vs. neutral: 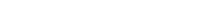).

**Figure 7:**
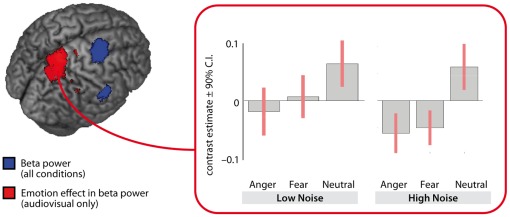
Source localization of beta–suppression. In the left panel, localized overall beta–suppression is depicted in blue (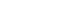, family-wise error corrected). The difference between emotional and neutral audiovisual beta-power localizes to a more posterior area (shown in red, 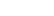, uncorrected). This difference is also seen in the contrast estimates (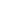
90% confidence intervals) shown in the right panel for both, high and low levels of noise.

When localizing the origin of the beta-suppression across all conditions (see Methods) we found the right premotor cortex as the primary source (Figure 7; Z = 6.42, MNI peak coordinate 24, −3, 46; BA 6). Second, the differential beta-suppression for emotional compared to neutral stimuli in the audiovisual conditions seems to originate from the more posterior superior parietal cortex (Z = 4.19, MNI peak coordinate 14, −54, 78; BA5). The condition-wise power changes as obtained from this posterior parietal source (Figure 7) show that difference between emotional and neutral stimuli can be seen in both, high and low noise levels.

## Discussion

Using ecologically valid stimuli we investigated the integration of body and vocal expressions in emotional communication. Body expressions show a clear influence on the processing of vocalizations leading to facilitated processing within less than 200 ms. Importantly, a modification of this influence by signal quality suggests integration of both modalities (rather than mere interaction) according to the IE principle [10]. Furthermore, emotional content elicits enhanced signal processing irrespective of the accompanying vocalization, as indicated by a stronger beta-suppression for visual as well as audiovisual stimuli.

### Interaction between voice and body expressions

On a behavioral level, we observed a clear improvement in performance, when visual and auditory information was present, in comparison to both purely auditory conditions. The participants showed the worst discrimination ability in the unisensory auditory condition with high levels of noise, replicating the results from our pilot study and demonstrating that intelligibility was manipulated as intended.

As predicted, we observed a clear interaction of auditory and visual information in the auditory N100 time-range. Previous studies have reported a facilitatory influence of concurrent visual information on early auditory perception [30]. While we were able to show in a previous study [7] that such an influence also occurs in early multisensory communication, we now extend this finding by demonstrating that such facilitation can be observed even in the absence of facial information.

Processing advantages for emotional auditory information have been observed before at slightly later (P200) processing stages [26], [49]. However, our results imply that auditory emotion processing can be further sped–up by reliable (i.e. predictive) body motion information. Stekelenburg and Vroomen [30] suggest that this early facilitatory influence through visual information may be mediated by the simulation of actions in motor areas such as the premotor cortex [50], an assumption supported by the localization of the beta-suppression discussed below. Our results provide evidence that this influence not only allows for a precise prediction of the auditory onset, but that this prediction process is modulated by the emotional content of the signal.

### Integration of voice and body expressions

The second main goal of the present study was to investigate whether vocalizations and body expressions are truly integrated (in the sense of the IE principle) and if so, under which conditions. By manipulating the noise level of the auditory signal, we contrasted differences between auditory and audiovisual processing in conditions of different signal quality. In the auditory N100 time-window, we observed clear inverse effectiveness; only at a high but not at a low noise level, a multisensory combination of visual and auditory information causes a speed–up in the peak latency of the N100. In contrast, if the auditory signal quality is satisfying (i.e. low noise), additional information from body expressions offers little processing advantage. Hence, information from body expressions and vocalizations has to be integrated very early in order to differentially affect auditory processing dependent on the noise level.

While this effect is observed most vigorously in the N100 latency, observations in the time–frequency-domain (beta-band) point in the same direction. At central electrodes, a larger suppression was observed for emotional compared to neutral stimuli. This pattern was observed for high but not for low noise stimuli. Furthermore, this effect points in an interesting direction for future studies: Is audiovisual integration not only affected by exogenous factors such as noise but also modulated by endogenous or content factors of the signal? That is, are emotional stimuli – highly salient and possibly necessitating rapid action – integrated more efficiently and processed faster? This hypothesis receives support from several studies reporting enhanced multisensory integration for highly salient stimuli, such as looming signals, in animals [51], [52] but also in humans [53], [54]. So far, EEG signatures of multisensory integration in communicative settings have mainly been investigated in the mismatching paradigm [23], [55]. We show that the IE principle, which has been primarily applied in fMRI and neurophysiological research [17]–[20], [50], [56] offers an alternative approach which not only reliably indicates integration but at the same time maintains ecological validity of the stimulus material. IE in the EEG signal has so far been demonstrated only in one recent study by Senkowski et al. [21] who used very simplified stimulus material consisting of Gabor patches and sinusoidal tones. The present data show that IE can be observed in the EEG for more complex, naturalistic stimuli. While it remains debatable at the current state of research whether the IE principle, originating from neurophysiology, can be directly applied to more indirect, noninvasive measures such as EEG (or also fMRI) [57], we believe that it is a promising approach in need of further investigation. With the present study, we take a step in this direction.

As outlined in the introduction, our study differs from most previous studies on ecologically valid multisensory integration [8], [9], [22] in that the immediate source of the sound (i.e. the mouth) is not visible. Yet, the relation between visual and auditory stimuli in the present study is not arbitrary, as the voice is a direct product of body movements. An interesting question for future studies is therefore the comparison between multisensory integration of information from face and voice with that from body and voice. Does the more direct link between face and voice increase observed integration effects? Or is the biological source of information present in both settings sufficient to elicit optimal integration?

### Processing emotional body expressions

Finally, we were interested in the processing of body expressions per se. While suppressed oscillatory power in the beta-range has been commonly observed in the perception of biological motion [32], [33], [58], we extend these findings to a modulatory effect of emotional content on the processing of body expressions. This observation is especially relevant as it has been suggested that beta as a correlate of sensory processing and attention, on the one hand, and the mu rhythm (in the same frequency-range) as a correlate of social processing, on the other hand, are distinct [59]. To differentiate between these functions, a separate analysis of occipital (sensory beta) and central electrodes (mu) has been proposed [33], [60]. Our emotion effect for visual and audiovisual conditions was only observed at occipital electrodes, which could suggest that the observed differences may be mainly driven by physical differences in the stimulus material.

A stronger beta-suppression for audiovisual compared to auditory stimuli is in line with previous findings reporting stronger suppression of beta in the perception of biological motion [61], [62]. Localization of the overall beta-suppression – irrespective of the experimental condition – yielded a source in the premotor cortex. Concomitantly, previous fMRI studies report activation in the premotor cortex for the perception of biological motion [63]–[65]. The premotor cortex has also been suggested to play a key role in the perception of emotional body expressions [66]. Thus, this localization of beta-suppression contrasts with a purely sensory interpretation of effects in the sensor space and rather suggests processes specific to the perception of biological motion. Hence, most likely, the observed beta effects arise from a combination of sensory and biological-motion-specific processes. The exact contributions of these processes will have to be delineated in future studies. Another notable result is the localization of emotion-specific beta-band differences in the right posterior superior parietal cortex in audiovisual emotion perception. This localization is also consistent with the localization results of beta-suppression during biological motion perception in previous studies [61], [62]. It appears that multisensory emotional input is able to elicit differences in areas specifically involved in the processing of biological motion rather than in unisensory visual or auditory areas only. This is another piece of evidence for a modulatory effect of affective information on biological motion processing. Future studies employing higher spatial resolution (fMRI, but also magnetoencephalography to retain temporal resolution) and different experimental designs will be necessary to determine cortical and subcortical loci of an interaction between biological motion and emotional vocalizations more precisely. However, our study provides a valuable starting point by highlighting differences in data interpretation depending on the analysis technique (sensor level versus source space).

### Conclusions

Humans rapidly integrate information from various sources, a crucial pre-requisite for successful social interaction. We are able to show that the perception of body expressions is influenced by emotional content of these expressions as well as by accompanying vocalizations. While previous studies have focused on the role of body expressions in multisensory communication in mismatch paradigms [6], [25], we show that in congruent settings, interaction effects similar to face–voice interactions can be observed. Furthermore, we provide evidence that the inverse effectiveness principle can be used to investigate multisensory integration in EEG data. It provides a valuable tool to study the neural processing of complex, ecologically valid information. Finally, we show that ERPs and neural oscillations, when used complimentarily in the investigation of multisensory integration, allow for a more comprehensive understanding of the interplay between different modalities.
